# Targeted Muscle Reinnervation for Trauma-Related Amputees: A Systematic Review

**DOI:** 10.7759/cureus.28474

**Published:** 2022-08-27

**Authors:** Zachary W Fulton, Benjamin C Boothby, Seth A Phillips

**Affiliations:** 1 Orthopaedic Surgery, Mercy Health Saint Vincent Medical Center, Toledo, USA

**Keywords:** residual nerve pain, neuroma, phantom limb, trauma, amputation

## Abstract

While amputation techniques have improved over time, questions remain around how to best treat neuromas and severed nerves in the amputee population, specifically for trauma-related amputees. This systematic review investigates and summarizes outcomes following targeted muscle reinnervation (TMR) for the trauma-related amputee population. Studies were classified based on primary or secondary TMR and relevant outcomes, including the ability to use a prosthesis, post-TMR opioid use, Patient-Reported Outcomes Measurement Information System (PROMIS) scores for phantom limb pain and residual limb pain, and overall pain resolution/reduction. Following TMR for trauma-related amputation, most patients experienced neuroma pain resolution (86.2%, 95% confidence interval [CI]: 67.2-95.0%) and overall pain reduction/resolution (90.7%, 95% CI: 82.2-95.4%). No differences were seen between primary and secondary TMR. Preliminary evidence indicates that TMR is effective for preventing or treating pain in patients with trauma-related amputation, whether used in the acute or delayed setting.

## Introduction and background

Nearly two million people are living with a form of limb loss in the United States, with up to 45% of cases attributed to trauma [1]. This population is at risk of developing symptomatic neuromas within the residual limb. Wallerian degeneration and a loss of neurotrophic factors cause a directionless propagation of axons, fibroblasts, Schwann cells, and blood vessels [2-6], which present as a painful neuroma in approximately 25% of those undergoing amputation [7]. A tight prosthetic fit combined with a symptomatic neuroma can decrease the patient’s overall satisfaction, functional abilities, and quality of life [8,9]. 

In individuals who develop painful neuromas, the excision or transfer of efferent motor nerves can be exploited for the benefit of the host [10,11]. Targeted muscle reinnervation (TMR) is a procedure that transfers severed nerves to new target muscles for the purpose of amplifying the targeted muscle motor signal, potentiating control of active prostheses, and decreasing neuroma-related pain [2,12]. TMR may be performed as either a primary or secondary procedure (i.e., in the acute setting at the time of amputation or in the delayed setting). While amputation techniques have improved over time, questions remain around how to best treat neuromas and severed nerves in the amputee population, specifically for trauma-related amputations. This systematic review investigates and summarizes outcomes following TMR for the trauma-related amputee population.

## Review

A systematic review of the available literature was performed according to PRISMA guidelines [13] and registered with PROSPERO (CRD42020205046). PubMed, Embase, and Cochrane databases were searched for relevant articles published from January 1, 2001, to April 2, 2021 (Table 1). References were reviewed for additional articles. To be included, articles had to be published in English and present primary clinical research about TMR for the adult (≥18 years) trauma population; if amputation reasons were mixed but the study provided patient-level data for trauma patients, the article was eligible for inclusion. Primary and secondary TMR procedures were included. TMR procedures were included if they were performed in either the upper or lower extremities. Studies were excluded if they were opinions/editorials, reviews/meta-analyses, or technical reports; non-human studies; case studies with less than five patients; research was for non-extremity amputation; amputation was due to illness; amputation reasons were mixed and there was insufficient patient-level data for inclusion; or were not relevant to the topic. Two independent reviewers screened articles for inclusion; any disagreements were discussed and resolved by the senior author (S.P.).

**Table 1:** Relevant articles published from January 1, 2001, to April 2, 2021. Number of results returned for each search term in PubMed, Embase, and Cochrane.

		PubMed	Embase	Cochrane
1	(targeted muscle reinnervation OR TMR) AND (trauma OR traumatic) AND (visual analog scale OR phantom limb pain)	9	10	3
2	(targeted muscle reinnervation OR TMR) AND (trauma OR traumatic)	72	25	10
3	("targeted muscle reinnervation" OR "TMR") AND ("trauma" OR "traumatic")	30	11	6
4	(targeted muscle reinnervation OR TMR) AND (trauma OR trauma amputee OR traumatic injury)	70	11	9
5	"targeted muscle reinnervation" AND (traumatic injury OR amputee) AND (phantom limb pain OR visual analog scale OR walking distance OR quality of life OR complications)	26	12	5
6	"targeted muscle reinnervation" AND trauma amputee	11	3	1
7	"nerve transfer" AND ("limb" or "extremity") AND (trauma amputee OR traumatic amputee OR traumatic injury) AND (visual analog scale OR phantom limb pain)	6	1	0
8	"nerve docking" AND ("limb" or "extremity") AND (trauma amputee OR traumatic amputee OR traumatic injury)	2	0	0

Outcomes assessed included ability to use a prosthesis, Patient-Reported Outcomes Measurement Information System (PROMIS) and numerical rating scale (NRS) scores for phantom limb pain and residual limb pain, opiate use pre- and post-TMR, and overall pain resolution/reduction. Risk of bias assessment was carried out for all eligible studies according to the Joanna Briggs Institute (JBI) template, which assesses for potential risk of bias based on 10-12 different domains according to the indicated study type [14]. Studies were scored and given an overall assessment of the low, moderate, or high risk of bias by two independent reviewers. A third reviewer adjudicated any differences and made final recommendations for study inclusion or exclusion.

Outcomes were summarized across studies with frequency counts and pooled rates, presented as percentages and 95% confidence intervals (CIs). Before analysis, raw proportions from individual studies were logit transformed. Pooled rates from logit transformed proportions were estimated using a random-effects model with inverse variance weighting and using the DerSimonian-Laird estimator for the estimation of the between-study variance component [15]. The corresponding 95% CIs were computed using normal approximation from logit transformed proportions. As appropriate, the Haldane-Anscambe correction was conditionally applied to correct zero-cell counts [16,17]. After computing pooled rates and corresponding 95% CIs, logit transformed estimates were exponentiated to aid in interpretation.

**Figure 1:**
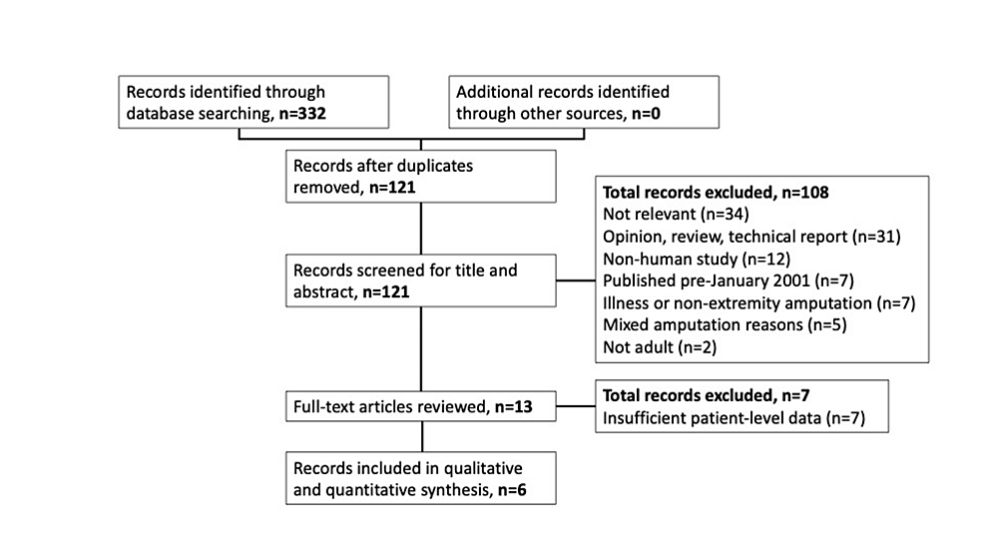
Flow diagram of the search results after inclusion and exclusion criteria were applied.

A total of 332 articles were identified in the initial literature search, with 121 remaining after duplicates were removed. Of the 121 articles, 108 were excluded based on title and abstract. Thirteen full-text articles were reviewed, of which seven articles were excluded for insufficient patient-level data. This left six total studies meeting the criteria for inclusion (Figure 1). Two studies were low [3,18], three studies were moderate [5,6,19], and one study was considered at high risk of bias [20] according to the JBI assessment; additional details are available in Tables 2, 3.

**Table 2:** Internal validity assessment of selected case series.

Study Information	Internal Validity										Overall Assessment of the Study					
Author	Were there clear criteria for inclusion in the case series?	Was the condition measured in a standard, reliable way for all participants included in the case series?	Were valid methods used for identification of the condition for all participants included in the case series?	Did the case series have consecutive inclusion of participants?	Did the case series have complete inclusion of participants?	Was there clear reporting of the demographics of the participants in the study?	Was there clear reporting of clinical information of the participants?	Were the outcomes or follow-up results of cases clearly reported?	Was there clear reporting of the presenting site(s)/clinic(s) demographic information?	Was statistical analysis appropriate?	% Yes	Risk of bias	Assessment	Assessment after contacting study authors	Level of evidence	
Frantz et al. [3]	Yes	Yes	Yes	Yes	Unclear	Yes	Yes	Yes	No	Yes	80	Low	Include	Include	4c	
Janes et al. [20]	Yes	No	Yes	Unclear	Unclear	No	No	No	No	NA	20	High	Include	Include	4c	
Souza et al. [18]	Yes	Yes	Yes	Yes	Yes	Yes	Yes	Unclear	No	Yes	80	Low	Include	Include	4c	
Pet et al. [19]	Yes	Yes	Yes	Unclear	No	Yes	Yes	Unclear	No	Yes	60	Moderate	Include	Include	4c	

**Table 3:** Internal validity assessment of selected quasi-experimental studies.

Study Information	Internal Validity									Overall Assessment of the Study					
Author	Is it clear in the study what is the "cause" and what is the "effect" (i.e. there is no confusion about which variable comes first)?	Were the participants included in any comparisons similar?	Were the participants included in any comparisons receiving similar treatment/care, other than the exposure or intervention of interest?	Was there a control group?	Were there multiple measurements of the outcome both before and after the intervention/exposure?	Was follow-up complete and, if not, were differences between groups in terms of their follow-up adequately described and analyzed?	Were the outcomes of participants included in any comparisons measured in the same way?	Were outcomes measured in a reliable way?	Was appropriate statistical analysis used?	% Yes	Risk of bias	Assessment	Assessment after contacting study authors	Level of evidence	
O'Brien et al. [5]	Yes	Unclear	Unclear	Yes	No	Yes	Yes	Yes	Yes	67%	Moderate	Include	Include	2d	
Valerio et al. [6]	Yes	Yes	Unclear	Yes	No	No	Yes	Yes	Yes	67%	Moderate	Include	Include	2d	

Three studies had data exclusively for primary TMR [3,5,6], one study examined only secondary TMR [18], and two studies examined both [19,20]. Study characteristics (including sample size, mean age, and sex) are shown in Table 4. The most commonly reported outcomes, and the only outcomes reported in greater than two articles, were neuroma pain resolution and overall pain reduction or resolution (including phantom limb pain). Other outcomes reported in greater than one study included the ability to use a prosthesis, post-TMR opioid use, and PROMIS scores for phantom limb pain and residual limb pain.

**Table 4:** Characteristics of the included studies. Data are reported as n/N (%), mean ± standard deviation, or as mean (range). --, data not available for trauma-related TMR population; F, female; M, male; TMR, targeted muscle reinnervation. *Primary TMR. **Secondary TMR. †Primary and secondary TMR. ‡Parentheses represent interquartile range rather than range.

	Sample Size	Sex (F)	Age (Years)	Follow-up (Months)
Janes et al. [20]^†^	17	--	--	--, (1-14)
Souza et al. [18]**	26	4/26 (15.4%)	32.8 ± 11.7	27.6 ± 27.5
Pet et al. [19]^†^	12*	2/12 (16.7%)	34 (14-59)	22 (8-60)
	23**	8/23 (34.8%)	44 (20-80)	22 (4-72)
Frantz et al. [3]*	25	10/25 (40.0%)	47.5 ± 13.1	14.1 ± 7.6
Valerio et al. [6]*	16	--	--	--
O’Brien et al. [5]*	6	--	--	23.6 (11-23)^‡^

Based on the overall reporting of neuroma pain resolution in 30/34 (88.2%) included patients, the pooled mean estimate for neuroma pain resolution was 86.2% (95% CI: 67.2-95.0%); there were no appreciable differences between pooled mean estimates for primary TMR (91.7%, 95% CI: 58.7-98.8%) and secondary TMR (84.2%, 95% CI: 49.9-96.6%). Studies reported overall pain prevention, reduction, or resolution in 79/85 (92.9%) included patients, with a similarly high pooled mean estimate of 90.7% (95% CI: 82.2-95.4%). Likewise, there were no appreciable differences in estimated rates between primary TMR (91.4%, 95% CI: 78.0-96.9%) and secondary TMR (90.1%, 95% CI: 76.4-96.3%) (Table 5).

Frantz et al. and Souza et al. reported the ability to use a prosthesis; their pooled mean estimate indicated that 81.8% (95% CI: 65.9-91.3%) of patients were able to use a prosthesis after TMR. For post-TMR opioid use, Frantz et al. and Valerio et al. collectively found that an estimated 13.2% (95% CI: 5.6-28.1%) of patients used opioids at the latest follow-up (Table 5).

**Table 5:** Outcomes following targeted muscle reinnervation. Data are reported as n/N (%). --, data not available for traumatic amputation TMR population; TMR, targeted muscle reinnervation. *Primary TMR. **Secondary TMR. †Primary and secondary TMR. ‡Counts (n/N) were back-calculated rather than provided outright.

	Neuroma Pain Resolution	Pain Resolution or Reduction	Ability to Use Prosthesis	Post-TMR Opioid Use
Janes et al. [20]^†^	5/7 (71.4%)	10/10 (100.0%)	--	--
Souza et al. [18]**	14/15 (93.3%)	15/15 (100.0%)	23/26 (88.5%)	--
Pet et al. [19]^†^	11/12 (91.7%)	31/35 (88.6%)	--	--
Frantz et al. [3]*	--	23/25 (92.0%)	19/25 (76.0%)	4/25 (16.0%)
Valerio et al. [6]*	--	--	--	1/16 (6.3%)^‡^

O’Brien et al. and Frantz et al. reported behavior, intensity, and interference PROMIS scores for phantom limb pain and residual limb pain. O’Brien et al. reported scores that fell below 50 in each domain, which is the normative mean for that measure in the United States (standard deviation = 10) [21]. Frantz et al. reported PROMIS scores as raw numerical values, precluding comparison to O’Brien, although there were some differences observed based on sex (Table 6).

**Table 6:** Phantom limb and residual limb pain. Data are reported as median (interquartile range). Bolded values were significantly different. F, female; M, male; TMR, targeted muscle reinnervation. *Primary TMR.

	Sample Size	Phantom Limb Pain			Residual Limb Pain		
		Behavior	Intensity	Interference	Behavior	Intensity	Interference
Frantz et al. [3]*	15 (M)	15 (14-16)	5 (4-7)	8 (8-11)	14 (7-17)	5 (3-7)	8 (8-10)
	10 (F)	7 (7-15)	3.5 (3-4.25)	8 (8-8)	7 (7-7)	3 (3-4)	8 (8-8)
O’Brien et al. [5]*	6	45.8 (36.7-55.6)	35.5 (30.7-40.2)	45.3 (40.7-51.2)	44.9 (36.7-54.8)	35.5 (30.7-43.5)	44.3 (40.7-54.1)

The treatment algorithm for amputations has changed drastically over the years, with new treatments and advancing prostheses now available to patients. TMR is one advancement that may lead to amputation procedures becoming less painful, more functional, and more cost-effective than limb salvage. In this systematic review of TMR in the trauma-related amputee population, there was a high rate of neuroma pain prevention, reduction, and resolution. There was a similar, and certainly associated, high rate of overall pain resolution or reduction found in this study. Notably, no differences were observed between TMR as a primary or secondary procedure for either of these outcomes. Prosthetic wear rates were also high in this study, while post-TMR opioid use was low. All these data points indicate that TMR is a promising procedure that deserves wider consideration in the traumatic amputee population.

TMR can improve residual limb muscle mass and reduce pain-causing sporadic signal firing by giving the nerve “somewhere to go, and something to do” [6]. Intuitively, if a patient has less pain, they will be more apt to wear a prosthesis on the residual limb. Moreover, the patient could wear a tighter-fitting prosthesis that is more functional or be fitted with a myoelectric prosthesis. These interconnected, tiered effects of TMR were observed in the present study; in addition to high rates of the neuroma, phantom limb, and residual limb pain reduction/resolution, prosthetic wear rates after TMR were also high in this review. Prior studies of amputation without TMR have reported a 27-56% prosthesis wear rate for the upper extremity and 49-95% for the lower extremity [22], whereas the estimated rates in the present study were consistently on the uppermost end of these ranges. Importantly, one of the studies reporting prosthesis wear rates exclusively performed amputation at the transhumeral and transradial level [18], while the other study did not stratify outcomes based on upper- or lower-extremity amputation [3]. Additionally, the relatively small number of included patients in these studies should be considered. However, the evidence in this review collectively supports the efficacy of TMR to not only improve neuroma, phantom limb, and residual pain but also improve postoperative limb functionality via prosthetic wear rates.

Although post-TMR opioid use was generally low in this review, it was not always significantly lower postoperatively compared to preoperatively, which is likely multifactorial. For example, in Frantz et al. [3], eight trauma patients reported preoperative opioid use. Four of the eight patients who used opioids preoperatively were able to stop by five months, while the remaining patients had not stopped by final follow-up. Predictably, the outlook was slightly improved for those who did not use opioids preoperatively; all the patients in Frantz et al.’s study who did not use opioids before the operation had stopped by one year after the operation, and at a mean of two months quicker than patients on prescription opioids prior to the operation. Many trauma patients take chronic opioid and neurotropic agents for several months to years prior to surgery or have psychological factors that implicate continued use [23,24]. The results from this study illustrate the influence of preoperative opioid use on postoperative opioid use in the trauma population undergoing TMR.

In a larger study by Valerio et al. [6], post-TMR opioid use for trauma- and non-trauma-related amputee patients was 61%, 37%, and 21% at six weeks, three months, and one year, respectively. However, when examining the trauma population independently, post-TMR opioid use was essentially unchanged, from 9.1% before the operation to 10.0% one year after the operation. Post-amputation pain may have multiple etiologies that are difficult to control for, including sharp bone edges, poor soft tissue coverage, painful scars, and ischemic pain [5,25]. These conditions are not correctable with TMR and may lead to prolonged chronic opioid use. Given the importance of pain reduction in this population and the risks of chronic opioid use, there should be a continued search for solutions that reduce or resolve pain in amputee patients while minimizing the need for postoperative opioid use.

This review adds to the growing literature that supports the use of TMR in the acute and delayed setting. In perhaps the only randomized controlled trial to directly compare TMR with traditional treatment (neurectomy and muscle burying), Dumanian et al. [26] found that those undergoing TMR reported a mean decrease in worst phantom limb pain that was 3.4 points greater on an 11-point NRS scale (0-10) one year after operation; this difference was significant on longitudinal mixed-model analysis. Differences in residual limb pain decrease between treatment groups approached significance and favored the TMR group. In a related single-arm prospective study, Mioton et al. [27] again found that TMR significantly reduced phantom limb pain and residual limb pain after amputation. Additional research is needed to confirm these results and guide TMR treatment algorithms for potentially eligible patients.

What is yet to be identified is the additional TMR surgical time commitment added to the traditional amputation, and how that affects other patient outcomes such as complication rates. Primary amputations in the trauma population often need to be performed quickly, sometimes emergently, due to hemodynamic compromise or other factors [28,29]. Incorporation of TMR for amputees of this specific population may therefore depend on the situation, surgeon skillset, and/or facility. Despite the complexities of treating patients with traumatic injuries, TMR shows promise and may substantially benefit the amputee population, including those undergoing amputations related to trauma.

TMR is a relatively new technique in the field of orthopedic traumatology, so there are limited data encompassing this niche of patients. Because of the limited data on this topic, this review did not have stringent requirements in terms of study design; of the six studies meeting inclusion criteria in this review, five studies were retrospective case series or cohorts, and only one was prospective. Additionally, each study had low power and limited ability to compare intervention and control groups without violating selection bias. Studies include several different amputation levels, body regions, operative periods, and surgeons performing the operation, all of which can influence outcomes. There are also various TMR techniques, so more research is needed on nerve transfer patterns and their effect on postoperative outcomes. Finally, some PROMIS scores and other outcomes were difficult to compare between studies due to variability in scoring systems, forms, and reporting.

## Conclusions

TMR is a technique used for amputees that can benefit neuroma pain, phantom limb pain, residual limb pain, and potentially narcotic use and prosthetic wear. TMR performed in the acute or delayed setting in this population appears to have equally improved outcomes related to pain and function. Limited high-quality TMR studies have been performed on the trauma population. This area would significantly benefit from further investigation.
